# Genetic Factors Mediate the Impact of Chronic Stress and Subsequent Response to Novel Acute Stress

**DOI:** 10.3389/fnins.2019.00438

**Published:** 2019-05-21

**Authors:** Elena E. Terenina, Sonia Cavigelli, Pierre Mormede, Wenyuan Zhao, Cory Parks, Lu Lu, Byron C. Jones, Megan K. Mulligan

**Affiliations:** ^1^GenPhySE, ENVT, INRA, Université de Toulouse, Castanet-Tolosan, France; ^2^Department of Genetics, Genomics and Informatics, University of Tennessee Health Science Center, Memphis, TN, United States; ^3^Department of Biobehavioral Health, Pennsylvania State University, University Park, PA, United States

**Keywords:** stress, hippocampus, microarray, C57BL/6J, DBA/2J, C57BL/6NJ

## Abstract

Individual differences in physiological and biobehavioral adaptation to chronic stress are important predictors of health and fitness; genetic differences play an important role in this adaptation. To identify these differences we measured the biometric, neuroendocrine, and transcriptional response to stress among inbred mouse strains with varying degrees of genetic similarity, C57BL/6J (B), C57BL/6NJ (N), and DBA/2J (D). The B and D strains are highly genetically diverse whereas the B and N substrains are highly similar. Strain differences in hypothalamic-pituitary-adrenal (HPA) axis cross-sensitization were determined by plasma corticosterone (CORT) levels and hippocampal gene expression following 7-weeks of chronic mild stress (CMS) or normal housing (NH) and subsequent exposure to novel acute restraint. Fecal CORT metabolites and body and organ weights were also measured. All strains exposed to CMS had reduced heart weights, whereas body weight gain was attenuated only in B and N strains. Acute stress alone produced larger plasma CORT responses in the D and N strains compared to the B strain. CMS paired with acute stress produced cross-sensitization of the CORT response in the N strain. The N strain also had the largest number of hippocampal transcripts with up-regulated expression in response to stress. In contrast, the D strain had the largest number of transcripts with down-regulated expression following CMS and acute stress. In summary, we observed differential responses to CMS at both the physiological and molecular level among genetically diverse strains, indicating that genetic factors drive individual differences in experience-dependent regulation of the stress response.

## Introduction

Stress is a generic term used to describe physiological and behavioral responses to real or perceived challenges (Wang et al., 2013). These responses are integrated over multiple systems (e.g., autonomic, behavioral, endocrine, and immune) with the hypothalamic-pituitary-adrenal (HPA) axis being a main pillar of the neuroendocrine response to stress. Ultimately, stimulation of the HPA axis results in glucocorticoid hormone [corticosterone (CORT) in mice and cortisol in humans] release. Glucocorticoids act on a wide range of cells and tissues via glucocorticoid and mineralocorticoid receptors, and control numerous metabolic pathways, immune system and brain functions, and also exert strong negative feedback on HPA axis activation (Dallman et al., 1994; Chrousos, 1998). Negative feedback regulation by glucocorticoids occurs both rapidly and over a longer time scale affected by previous stress experience (Myers et al., 2012). Rapid regulation occurs within minutes through direct inhibition of neurons in the hypothalamic paraventricular nucleus (PVN). In contrast, delayed and experience-dependent negative feedback is partly mediated by glucocorticoid receptors in limbic brain regions, including the hippocampus, that project indirectly to the PVN. The hippocampus is a key negative regulator of HPA axis activity based on previous stress exposure and exhibits structural and functional alterations following chronic stress as well as distinct gene expression changes in response to acute or chronic stress (McEwen, 1999). The critical inhibitory role of this structure on HPA axis activity has been demonstrated through biological and genetic deletion studies (van Haarst et al., 1997; Furay et al., 2006; Herman and Mueller, 2006).

Appropriate activation and negative feedback regulation of the HPA axis following acute stress is critical for short-term survival; however, prolonged activation of the HPA axis in response to chronic stress can have adverse effects on health (Kessler, 1997; Hammen, 2005; Ohman et al., 2007). Individual differences in HPA axis function are well-documented (Mormede et al., 2002; Mormede et al., 2011). Individuals also vary in their particular susceptibility to the effects of chronic stress on health (McEwen and Stellar, 1993; McEwen, 1998) and behavior (Vitousek et al., 2014). Importantly, some individuals may be more prone to developing stress-related disorders while others may be insensitive or display greater resilience to the effects of stress (Wood et al., 2010; Nasca et al., 2015). In summary, genetic variation in HPA axis regulation likely mediates individual differences and vulnerability to health risks associated with chronic stress. Here, we test the hypothesis that prior stress experience produces genotype dependent changes across multiple system levels, including hippocampal transcription, HPA axis regulation, and organ physiology. Moreover, these genetic differences in response to chronic stress also alter the response to subsequent stress-challenge.

To evaluate individual genetic differences in stress response, we assessed biometric data, HPA axis activity, and hippocampal gene expression among three inbred mouse strains exposed to chronic and acute stress. The strains used in this study, C57BL/6J (B), C57BL/6NJ (N), and DBA/2J (D), were selected because they differ in both genetic background and stress response. B and D strains are polymorphic at ∼5 million loci (SNPs and small indels) (Wang et al., 2016), demonstrate marked behavioral differences in response to stress (Mozhui et al., 2010; Urquhart et al., 2016), and represent the parental strains of the well-characterized BXD recombinant inbred panel. The BXD panel has been used to assess the role of chronic stress on cognitive performance (Jung et al., 2017), learning and memory (Shea et al., 2015), and emotional behavior (Carhuatanta et al., 2014) in a diverse genetic population. B, D, and their recombinant inbred BXD progeny, differ in stress response and underlying hippocampal transcriptional mechanisms; however, genetic factors driving differences in HPA regulation among the BXD strains have yet to be determined.

In contrast to the divergent genetic background of the B and D strains, the B and N substrains are closely related. In fact, the two strains trace back to a common inbred C57BL/6 stock in 1952. Over the intervening decades the substrains have accumulated about 20K SNP and small indel variants across the genome. This level of variation is about 0.4% of that between B and D strains (Keane et al., 2011). Despite their common origin and highly similar genetic composition, the B and N substrains differ markedly across a surprisingly large number of phenotypes (Mulligan et al., 2008; Matsuo et al., 2010; Simon et al., 2013; Veeraiah et al., 2014). Males of the N substrain display more depression-like behavior following chronic CORT treatment compared to the B strain (Sturm et al., 2015). The mechanisms underlying different physiological and biobehavioral responses to stress between substrains are unknown.

In the present work we dissect the architecture of the response to stress using females from the B, N, and D strains and an innovative experience-dependent stress paradigm in which response to an acute novel stressor is measured following a history of normal housing (NH) or chronic stress exposure. Only female mice were included in the analysis because they are classically understudied in the field of stress biology, and there is evidence that females are more susceptible to HPA axis sensitization following chronic stress than males (Bangasser et al., 2010). Endpoint measures include plasma and fecal glucocorticoid levels, body and organ weights, and hippocampal gene expression. We report profound differences in the physiological and molecular response to chronic stress – even between females of the B and N strains that have highly similar genomes – and identify genes and biological pathways that may underlie individual variation in HPA axis regulation following chronic stress.

## Materials and Methods

### Animals

Female mice from three inbred mouse stains, C57BL/6J (B), C57BL/6NJ (N), and DBA/2J (D) were assigned to four groups: (1) NH, (2) chronic mild stress (CMS), (3) normal housing followed by acute restraint (NH-R), and (4) chronic mild stress followed by acute restraint (CMS-R). Numbers of mice per strain and treatment group ranges between 4 and 12 and are indicated in Supplementary Table 1. The majority of mice were purchased from the Jackson Laboratory and housed for at least 1 week prior to study enrollment, although a small number of B and D mice from a colony at the University of Tennessee Health Science Center were also included in the study (Supplementary Table 1). All procedures were approved by the University of Tennessee Health Science Center Institutional Animal Care and Use Committee (Institutional Protocol 14–131).

### Chronic Mild Stress, Acute Restraint and Blood and Organ Collection

During CMS, stressors were applied twice daily for 7 weeks, once during the light phase and once during the dark. Stressors consisted of four broad categories: light cycle changes, physical, social, and predator stressors (Supplementary Figure 1). Immediately following CMS, mice in the CMS and CMS-R group were returned to NH conditions for 1–4 days. Within this 4-day period, CMS-R, and NH-R groups were exposed to an acute stressor – 15 min of restraint in a ventilated 50-mL conical tube – followed by tail nick blood collection and euthanasia 45 min later. For all blood samples, plasma was isolated from whole blood and used to determine CORT concentration. Thymus, adrenals, heart, and brain were dissected, weighed, and flash frozen. Hippocampus was removed from the brain for gene expression analysis. Day of sacrifice over the 4-day period was counterbalanced across all strain and treatment groups and there was no effect of sacrifice day on any dependent measure.

### Corticosterone Analysis

To non-invasively estimate CORT circulation during the chronic stress protocol, fresh fecal samples were collected weekly by placing each animal into an empty cage and recovering all fecal pellets produced in an hour. CORT metabolites were extracted from feces and were measured in both plasma and fecal extracts using a commercial RIA kit (ImmuChem TM Double antibody 125I RIA kit from MP Biomedicals, Solon, OH, United States) that is supplied with a primary antibody that binds rodent CORT and metabolites (Cavigelli et al., 2005).

### Gene Expression

A total of four to five mice from each experimental group (Supplementary Table 1) were selected at random for analysis of hippocampal gene expression. Total RNA was extracted from frozen hippocampus using the AllPrep DNA/RNA Mini Kit (Qiagen). RNA was reverse transcribed into cDNA according to the manufacturers protocol followed by hybridization on the Affymetrix GeneChip Mouse Transcriptome Array 1.0 (Thermo Fisher Scientific, Santa Clara, CA, United States). Preparation of cDNA and microarray hybridization were completed at the Molecular Resource Center, a University of Tennessee Health Science Center Institutional Core, by experienced technicians. Affymetrix Expression Console Software was used to identify and remove outlier samples, normalize (RMA method) the resulting CEL files, and generate gene level log_2_ transformed expression profiles for 65,770 probe sets (transcripts). This list was further filtered to include 25,858 annotated gene transcripts used for downstream analysis. The full data set included hippocampal gene expression from 53 animals of three strains (B, N, and D) subjected to four different conditions (CMS, CMS-R, NH-R, or NH; Supplementary Table 1). Gene expression data is freely available for analysis or download at GeneNetwork.org^1^.

### Statistical Analysis

All statistical analyses were performed using the R software environment. Tissue weights are often adjusted using body weight as a covariate, however, body and organ weight were found to have a complex relationship in this study (Supplementary Table 2). Therefore, uncorrected raw tissue weights were used in the analyses. There was no effect of restraint on body or organ weight, thus subjects in the CMS and CMS-R groups were pooled (CMSgroup; numbers range between 16 and 20 per strain, Supplementary Table 1) and subjects in the NH-R and NH groups were pooled (NHgroup; numbers range between 12 and 14 per strain, Supplementary Table 1) for statistical analysis of the effect of CMS on these measures. Body and organ weights were analyzed using ANOVA (*Anova* function in the *car* package with type = 3 for Type III Sum of Squares calculation) for a two between-subject variable experiment that included strain (B, D, and N) and chronic stress treatment (CMSgroup or NHgroup). ANOVA effect size was calculated using Eta-squared η2 (Sum of Squares Between divided by Sum of Squares Total). *Post hoc* analyses of strain and/or treatment contrasts were performed using Welch’s unequal variance *t*-test. Fecal CORT metabolite concentration (fCORT) was measured weekly in the CMSgroup and log-transformed to meet assumptions of normality required for parametric statistical analyses and analyzed using a two-factor ANOVA (strain and time). Plasma CORT, measured in the CMS-R and NH-R groups after acute restraint and in the NH and CMS groups at the time of tissue harvest, was analyzed using a two-factor ANOVA that included strain (B, D, and N) and treatment (CMS or NH) following removal of outliers greater than 2 standard deviations from the mean (presumed technical artifact outliers removed within strain and treatment group). This resulted in the removal of 5 data points (D CMS-R; B CMS-R; N CMS-R; B NH-R; and B NH-R).

We predicted patterns of response for each transcript within strain as 0:1:0 or 1:1:0 (CMS:CMS-R:NH-R) for up-regulation of expression and as the inverse (1:0:1 or 0:0:1) for down-regulation of expression to acute stress following CMS. We then fit transcript expression within strain to each predicted pattern using a linear model and selected transcripts with a nominal *p*-value of 0.05 or less for further analysis. Correction for multiple testing (False Discovery Rate or FDR) was applied to the resulting *p*-values using the Benjamini-Hochberg (BH) procedure (Benjamini and Hochberg, 1995; Chipman and Tibshirani, 2006) but few transcripts had an FDR adjusted *p*-value less than 0.05. Therefore, we used the nominal *p*-value threshold for a descriptive analysis of neuroadaptive stress responses within and between strains. Transcripts were further prioritized to only include transcripts with significant (*p <* 0.05) mean expression changes greater than 10% between the NH-R and CMS-R group for each strain. A minimum expression threshold was applied as an additional filter because small effect size differences often prove difficult to reproduce across studies and expression platforms. This resulted in identification of 2,227 hippocampal transcripts (∼9% of detected genes) with stress responsive up- or down-regulation of expression in at least one strain.

### Functional Analysis

Enrichment analysis was performed using tools available at WebGestalt (Zhang et al., 2005; Wang et al., 2013). Target gene lists combined with a background gene set consisting of all 25,858 genes were used to identify enrichment terms. Microarray probe IDs were used as input for all lists. At least 5 genes per category and a significance level of less than 0.05 based on BH adjustment for multiple testing were used as criteria for significance.

## Results

### CMS Alters Fecal and Plasma CORT Concentrations in a Strain-Dependent Manner

**FIGURE 1:**
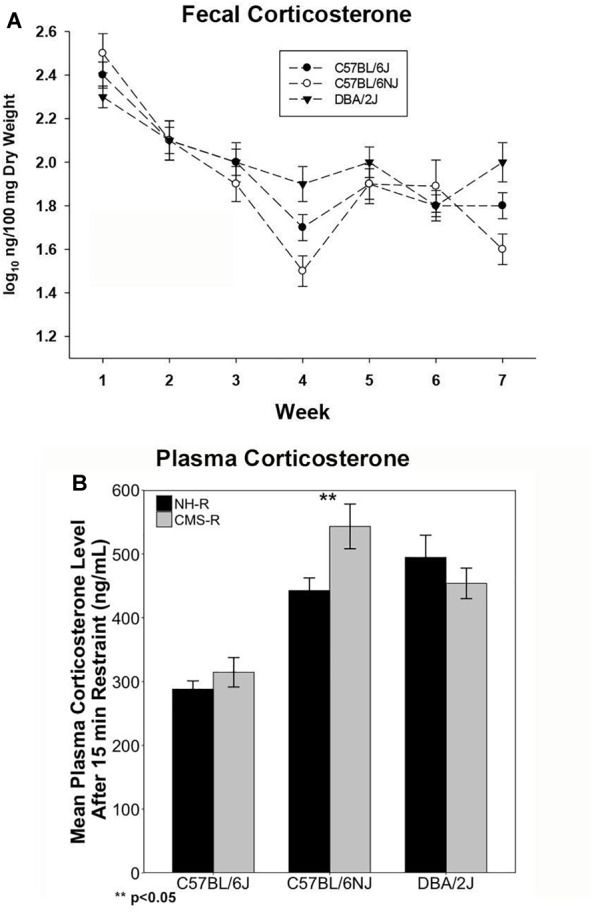
Corticosterone Measures. Fecal CORT (fCORT) during seven weeks of CMS is shown in **(A)** and plasma CORT measured at the end of 15-min restraint in normal housed (NH-R) and CMS treated (CMS-R) animals is shown in **(B)**. Habituation to CMS is observed as a return to basal fCORT levels in all three strains as shown in **(A)**. The D and N strain have a more pronounced CORT response to acute restraint stress compared to the B strain as shown in **(B)**. The N strain shows a sensitized CORT response to an acute novel stressor (15 min of restraint) following CMS. There is a significant main effect of strain (*p <* 0.001) and a significant strain-by-treatment interaction effect (*I* < 0.05) on plasma CORT response. Only the N strain shows a significant difference in CORT response based on chronic stress exposure (NH-R vs. CMS-R, *p <* 0.05).

We measured fecal CORT metabolite (fCORT) concentrations as a non-invasive estimate of glucocorticoid release and HPA regulation at baseline and during CMS treatment. There were significant differences in basal fCORT concentrations among the B (*n* = 14; 1.77 ± 0.20), N (*n* = 12; 1.73 ± 0.27), and D (*n* = 13; 1.93 ± 0.22) strains, with the D strain having higher fCORT levels at baseline compared to the B and N substrains (Supplementary Figure 2A). Weekly fCORT levels showed a strong time effect (*p <* 0.0001) with a steady decrease during the 7 weeks of CMS and a significant strain × week interaction (*p <* 0.01) (Figure 1A). The D strain had the lowest levels on week 1 (*p <* 0.05), and at the end of CMS treatment (week 7) there was a strong strain effect (*P <* 0.001; N, *p <* 0.05 and D, *p <* 0.07 compared to B). All strains demonstrated a return to baseline of fCORT levels by week 4.

To test the extent to which prior chronic stress resulted in changes in HPA axis regulation (sensitization or desensitization), we measured circulating plasma CORT concentrations following exposure to an acute novel stressor (15 min of restraint) in the CMS-R and NH-R groups (Figure 1B). There was a significant main effect of strain [*F*(2,46) = 20.35, *p <* 0.0001, η*^2^* = 0.88] and a significant strain-by-CMS interaction effect [*F*(2,46) = 3.48, *p <* 0.05, η*^2^* = 0.15]. Circulating CORT levels were significantly higher in N [*t*(24.208) = 6.84, *p <* 0.0001] and *D* [*t*(30.35) = 6.69, *p <* 0.0001] compared to B for all treatments (Figure 1B). Prior exposure to chronic stress produced significant enhancement of the CORT response to a novel acute stressor (cross-sensitization) in the N strain only [*t*(9.68) = 2.5, *p* = 0.03] (Figure 1B). Circulating plasma CORT levels were also measured at the time of tissue harvest for the NH and CMS groups and give an approximation of baseline CORT levels with or without CMS treatment. There was a significant main effect of treatment on time of death plasma CORT levels [*F*(1,29) = 6.4, *p* = 0.017] with higher plasma CORT in the B strain [*t*(8.92) = 2.36), *p* = 0.04] and a trend for higher CORT in the D strain [*t*(8.91) = 2.17), *p* = 0.06] associated with CMS treatment (Supplementary Figure 2B).

### Differential Impact of Chronic Stress and Strain on Body and Organ Weight Change

**FIGURE 2:**
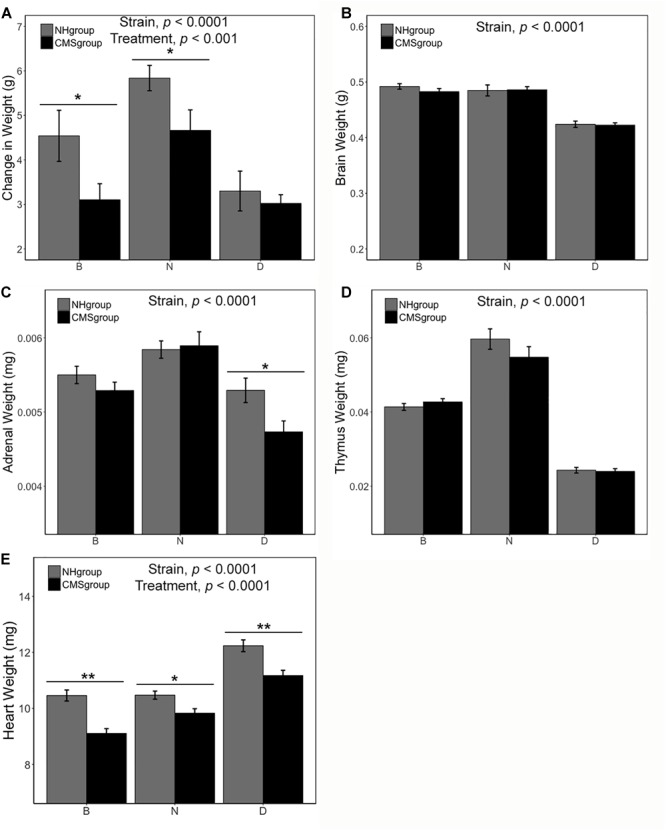
Body and organ weight measures following CMS treatment. Subjects in the CMS and CMS-R groups were pooled (CMSgroup; black) and subjects in the NH-R and NH groups (NHgroup; gray) were pooled for the analysis because there was no effect of restraint on body or organ weight. Difference in body weight between the end and beginning of chronic stress is significant for the B and N substrains with both strains showing less weight gain in the CMS group **(A)**. Following 7 weeks of CMS, tissue was harvested and weighed for all strains. Brain weight **(B)** was not altered by treatment but was significantly different between the D strain and both B and N substrains [D < B, *t*(49.25) = 12.32, *p* = 2.2e-12 and D < N, *t*(35) = 10.17, *p* = 5.46e-12]. Adrenal weight **(C)** was dependent on strain. The N strain had the largest adrenals [N > B, *t*(50.49) = 3.46, *p* = 0.001 and N > D, *t*(56.94) = 5.42, *p* = 1.27e-06] and the D strain had the smallest [D < B, *t*(54.43) = 2.82, *p* = 0.007]. CMS resulted in a reduction in adrenal weight only in the D strain. There was no effect of treatment on thymus weight **(D)**, however, there was an effect of strain. The N strain had the largest thymus [N > B, *t*(32.28) = 6.92, *p* = 7.45e-08 and N > D, *t*(30.52) = 15.65, *p* = 4e-16]. There was a significant main effect of treatment and strain on heart weight **(E)**. The D strain had the largest heart weight [D > N, *t*(53.73) = 7.28, *p* = 1.46e-09 and D > B, *t*(63) = 8.17, *p* = 1.8e-11]. Heart weight was also significantly different between the B and N substrains [N > B, *t*(57.27) = 2.1, *p* = 0.04]. CMS was associated with a significant reduction in heart weight in all strains. Significance criterion: ^∗^*p <* 0.05; ^∗∗^*p <* 0.01.

There was a significant main effect of strain [*F*(2,75) = 6.74, *p <* 0.0001, η*^2^* = 0.14] and exposure to CMS [*F*(1,75) = 6.43, *p <* 0.01, η*^2^* = 0.07] on body weight change (measured as the difference in weight between the end and beginning of CMS treatment, Figure 2A). The N strain gained more weight than both the B [*t*(50.83) = 3.14, *p <* 0.01] and D strain [*t*(37.23) = 4.98, *p <* 0.0001] over the seven weeks of the study (Figure 2A). However, CMS was associated with less weight gain overall [*t*(49.95) = 2.45. *p <* 0.05] (Figure 2A). Differences in weight due to CMS were significant for the B [*t*(16.24) = 2.12, *p* = 0.05] and N [*t*(21.81) = 2.17, *p <* 0.05] substrains (Figure 2A).

There was a significant main effect of strain on adrenal weight [*F*(2,87) = 17.44, *p <* 0.0001, η*^2^* = 0.27], brain weight [*F*(2,87) = 42.51, *p <* 0.0001, η*^2^* = 0.55], thymus weight [*F*(2,87) = 98.90, *p <* 0.0001, η*^2^* = 0.68], and heart weight [*F*(2,87) = 40.80, *p <* 0.0001, η*^2^* = 0.40] (Figure 2). The D strain had the smallest brain (*p <* 0.001) and biggest heart (D > N > B; all *p <* 0.05; individual contrast *p*-values given in Figure 2). In contrast, the N strain had the largest thymus (N > B > D; all *p <* 0.0001; individual contrast *p*-values given in Figure 2) and adrenals (N > B > D; all *p <* 0.01; individual contrast *p*-values given in Figure 2). There was a significant main effect of CMS on heart weight [*F*(1,87) = 29.85, *p <* 0.0001, η*^2^* = 0.15]. There was a trend for decreased adrenal weight (mean loss between NH and CMS groups of 0.0002 mg) following CMS [*t*(90.75) = 1.88, *p* = 0.06] (Figure 2C). Likewise, CMS was associated with an overall decrease (mean loss between NH and CMS groups of 10.4 mg) in heart weight compared to NH controls [*t*(84.6) = 4.52, *p <* 0.0001] and the effect was significant for all strains (Figure 2E; individual contrast *p*-values given in Figure 2).

### Chronic Stress Exposure Alters Hippocampal Transcriptional Response to an Acute Stressor in a Strain-Dependent Manner

**FIGURE 3:**
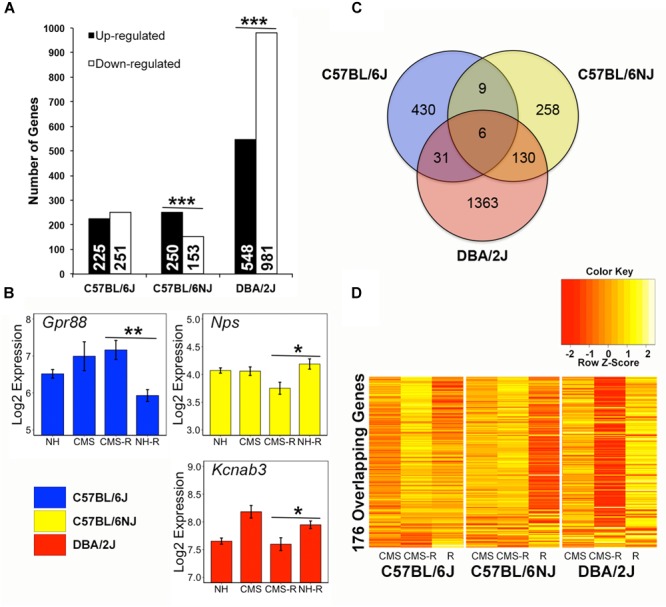
Strain differences in the hippocampal transcriptional response to stress. **(A)** Probe sets (transcripts) whose expression significantly (*p* < 0.05) fit stress up-regulated or stress down-regulated patterns (prioritized based on significant and greater than 10% fold change difference within strain between CMS-R and NH-R treatments) are categorized by strain and pattern. Chronic stress up-regulated transcripts have higher expression following combined chronic and novel acute stress treatment (CMS-R) compared to novel (NH-R) acute stress. Stress down-regulated transcripts have lower expression in the CMS-R treatment group compared to the NH-R treatment group. The N substrain shows a significantly higher number of transcripts up-regulated following stress compared to down-regulated transcripts. In contrast, the D strain has a significantly more transcripts whose expression is decreased in response to a novel acute stressor following chronic stress. No difference is observed for the B substrain. Significance determined using the Chi-Square test for independence. **(B)** Representative stress sensitive transcripts unique to B (blue), N (yellow), or D (red) are shown. *Gpr88* expression is up-regulated by acute stress following chronic stress exposure in B whereas *Nps* and *Kcnab3* expression is down-regulated by chronic stress in the N and D strain, respectively. Gpr88 is an orphan G protein-coupled receptor involved in glutamatergic and GABAergic signaling that may have a role in anxiety and learning and memory (Alavi et al., 2017) and has been previously identified as a chronic stress modulated gene in rodent hippocampus (Ubaldi et al., 2015). NPS encodes the stress sensitive neuropeptide S, which has been associated with regulation of feeding, fear response, and addiction-related traits (Schank et al., 2012). *Kcnab3* encodes a potassium voltage-gated channel subunit that is correlated with cognitive performance under chronic stress (Jung et al., 2017) in the BXD population derived from the B and D strains. **(C)** Comparison of strain specific expression among 2,227 probe sets showing up- or down-regulated expression following exposure to stress reveals strain overlap between 176 probe sets detected in two or more strains. **(D)** Hierarchical clustering of the 176 overlapping stress sensitive genes reveals contrasting expression between B and N substrains and the D strain. ^∗^*p* < 0.05; ^∗∗^*p* < 0.01; ^∗∗∗^*p* < 0.001.

The hippocampus is a critical mediator of experience-dependent negative feedback on HPA axis activation in response to stress. To explore HPA axis regulatory mechanisms that may drive individual differences in the response to chronic stress we identified probe sets (transcripts) with increased (up-regulated) or decreased (down-regulated) expression in the hippocampus in response to an acute stress challenge (restraint) following a history of chronic stress exposure. In total, 2,227 transcripts demonstrated an expression pattern consistent with CMS up- or down-regulation to a novel acute stressor (Figure 3A and Supplementary Table 3). Based on the chi-square distribution, significantly (*p <* 0.05) more transcripts were up-regulated vs. down-regulated by prior chronic stress in the N substrain (Figure 3A). In contrast, more transcripts were down-regulated vs. up-regulated by prior chronic stress in the D strain. The proportion of transcripts with increased or decreased expression in response to an acute stress challenge following CMS was roughly equal in the B strain. Examples of transcripts with up-regulated or down-regulated expression patterns that were significant and unique to a single strain are shown in Figure 3B and include *Gpr88, Nps*, and *Kcnab3*. Very little overlap was observed between strains; only 176 transcripts were detected in two or more strains (Figure 3C). For example, all nine transcripts detected in both the B and N substrains showed the same pattern of stress-induced up-regulation (*Kcnn3, Kcnk9, Sgsm1*, and *Mir344*) or down-regulation (*Srl, Orai2, Fam220-ps, Zcchc5*, and *Slain1os*) of expression. However, most overlapping transcripts between strains exhibited opposite patterns of stress-induced up- or down-regulation of expression between B and N substrains and the D strain (Figure 3D). For example, out of the 31 overlapping transcripts between the B and D strains, only 3 showed an effect in the same direction – decreased expression of *Fkbp5* (a negative regulator of glucocorticoid receptor function) and increased expression of *Mir6415* and *1700019G24Rik*. Likewise, of the 130 overlapping transcripts between the N and D substrains, only 15 displayed an effect in the same direction (Down-regulated: *Fn1, Fmo2, Mertk, Gkn3, Dbp, Hif3a, Tmc7, Paqr5, Ptprb, Ly6c1, Ly6c;* Up-regulated: *Nhlh2, Gimap6, Igkv4-58, 3110004A20Rik*). All six transcripts (*Prkar2b, Rgs11, Snhg11, Mir1906-2, Lrrc16b*, and *Mir5125*) detected in all three strains demonstrated stress-induced up-regulation of expression in both the B and N substrains and stress-induced down-regulation of expression in the D strain.

### Divergent Biological Processes Driving Strain Differences in Stress Response

To investigate underlying biological processes driving the divergent transcriptional response to chronic stress we examined enriched functional categories between sets of overlapping transcripts with opposite expression patterns (Supplementary Table 4). Transcripts with a significant and divergent stress response (up- vs. down-regulation) between the B and D strains were significantly enriched for localization to the synapse (*Asic1, Pcdh15, Grm8, Prkar2b, Lrrtm3*, and *Olfm2*) and dendrite (*Asic1, Prkar2b, Brinp3*, and *Rgs11*), and for heterotrimeric G-protein signaling pathways (*Grm8, Prkar2b, Rgs2*, and *Rgs11*). In contrast, transcripts divergent between the N and D strains were enriched for RNA processing (RNA and mRNA processing, metabolic process, splicing, and regulation of these processes), poly(A) RNA binding, and localization to the nuclear speck, nuclear body, spliceosomal complex, and mRNA surveillance pathway (Supplementary Table 4).

Enrichment analysis was also used to identify genes and biological processes represented by hippocampal transcripts detected in only one of the three strains with significant up- or down-regulation of expression in response to acute stress following CMS (Supplementary Table 5). Transcripts detected only in the B strain (430 transcripts), were significantly enriched for behavior (specifically, cognition, and learning and memory), neurogenesis and neuron projection development, synaptic signaling, synaptic localization, glutamate receptor activity, neuroactive ligand-receptor interaction, MAPK signaling pathway, and regulation of GTPase activity. In contrast, transcripts detected only in the D strain (1363 transcripts) were enriched for cell adhesion, localization to the extracellular matrix, negative regulation of signal transduction, enzyme-linked receptor protein signaling pathway, regulation of phosphorylation, localization to the cytoskeleton, regulation of RNA splicing, regulation of protein modification process, localization to the endoplasmic reticulum, lipid localization, and localization to the cell surface. In the N strain 258 transcripts were detected and were enriched for detection of a chemical stimulus, sensory perception (including sensory perception of smell), olfactory transduction, G-protein coupled receptor activity, and olfactory receptor activity.

## Discussion

### The Impact of Chronic Stress on Multiple Biological Systems Is Dependent on Strain

In this study we reported a differential impact of strain and chronic stress exposure on many different biological measures in females, including biometric body and organ weights, hippocampal transcriptional signatures, and glucocorticoid response. The impact of chronic stress on each measure was highly dependent on strain. Compared to controls, CMS was associated with a reduction in weight gain and heart weight in the B strain. Compared to the N and D strains, the B strain was the least responsive to the effects of CMS on hippocampal transcriptional and circulating CORT response to a novel acute stressor. However, enrichment analysis of the 430 hippocampal transcripts with CMS-induced up- or down-regulation of expression unique to the B strain revealed underlying biological processes relevant for biobehavioral adaptation to stress (cognition and learning and memory, synaptic signaling, synaptic localization, glutamate receptor activity, neuroactive ligand-receptor interaction, MAPK signaling pathway, and regulation of GTPase activity). In addition, CMS treatment in the B strain was associated with significantly higher circulating plasma CORT relative to NH controls. In contrast, the D strain demonstrated reduced heart weight and adrenal weight associated with chronic stress, greater circulating CORT responses to acute stress in general, and the largest hippocampal transcriptional response (mainly down-regulated) to an acute stressor following CMS. Similar to the B strain, CMS treatment in the D strain was modestly associated with higher circulating plasma CORT relative to NH controls. For the N strain, and similar to the closely related B substrain, CMS was associated with reduced weight gain and heart weight. Compared to the B substrain however, the N strain demonstrated greater circulating CORT responses to acute stress, was the only strain to show HPA axis cross-sensitization to acute stress after CMS, and had a larger hippocampal transcriptional response (mainly up-regulated) to an acute stressor following CMS. The N strain also demonstrated little difference in circulating plasma CORT between NH and CMS groups. It is feasible that these differences in stress response measures observed between female B, N, and D strains also drive differences in biobehavioral response to stress.

It is important to point out several limitations of our study. One such caveat is that measurement of behavioral response to CMS was not possible in the same cohort of animals tested for response to a novel acute stressor because exposure will alter subsequent behavior (and vice versa). Future studies that evaluate behavioral alterations in female B, D, and N strains following CMS are planned and will be required to understand how alterations in hippocampal gene expression and HPA axis reactivity modify the behavioral response to chronic stress in females. Of interest, related studies in male B and D strains also found cross-sensitization of the circulating CORT response to novel stressors in both strains, hippocampal gene expression changes related to excitatory neurotransmission in B males, and divergence in behavioral response (e.g., active response in B vs. passive response in D males) (Mozhui et al., 2010). Biobehavioral differences – increased food intake, decreased sucrose preference, and increased immobility in the forced swim test in N males relative to B males have also been observed following chronic elevation of CORT levels (Sturm et al., 2015). These studies suggest greater biobehavioral resilience to chronic stress in B males relative to D and N males, possibly through alterations in glutamatergic neuroplasticity (Mozhui et al., 2010). Although behavioral profiles in response to CMS have yet to be performed in B, N, and D females, our molecular and physiological response profiles suggest that the strain differences in biobehavioral response to CMS will be similar in females and we predict greater resiliency and more active response to stress in B females relative to D and N females. Previous studies have also reported more profound effects on chronic stress on behavior in females compared to males (Frisbee et al., 2015), however strain and stress protocols are inconsistent between studies and future experiments that include males and females along with matched strains and stress paradigms will be required to confirm these hypotheses.

A second limitation of our study is that we did not measure estrus cycle status in our female mice. The ability to detect significant main effects of stress and strain at multiple trait levels suggests that hormonal status is not a major cofactor in our study. However, the lack of estrus cycle status in our females precluded detailed analyses of both: (1) strain variation in hypothalamic-pituitary-gonadal axis function in response to stress, and (2) trait variation due to interactions between strain, stress, and hormonal level. For these reasons, and because the impact of individual housing and CMS on estrus cycle status in female B, N, and D mice is not well established, future studies are warranted. However, accurate estrus cycle monitoring requires multiple, possibly invasive, measurements over the duration of the study. The development of non-invasive and high-throughput methods for monitoring gonadal hormone status would greatly increase the feasibility of future studies into the impact of strain and estrus cycle variation on stress response.

### CMS and Cardiovascular Pathology

Heart weight was the only consistently altered biometric in our study. Remarkably, CMS was associated with decreased heart weight in B, N, and D female mice relative to controls. Our study was limited to heart weight, and the molecular mechanisms driving these changes remain elusive. However, in humans, stress has long been associated with cardiovascular disease and pathology. The CMS paradigm in rodents has high translational relevance for the study of interactions between genotype, stress exposure, and cardiovascular pathology, and previous studies have identified numerous cardiovascular changes following chronic stress (Grippo et al., 2006; Frisbee et al., 2015). However, ours is the first study to report cardiovascular alterations (decreased heart weight) following chronic stress in multiple strains of female mice. Future studies in genetic populations segregating B, D, or N alleles, such as the BXD population or a reduced complexity cross between B and N substrains (Kumar et al., 2013; Ashbrook et al., 2017; Kirkpatrick et al., 2017), will be needed to determine the precise genetic factors driving alterations in the cardiovascular system in response to chronic stress.

### Strain Differences in Hippocampal Transcriptional Response Implicate Excitatory Neurotransmission and Cell Signaling, Immune, and Metabolic Systems as Potential Drivers of Physiological and Behavioral Responses to Chronic Stress

In the current study, the majority of hippocampal transcripts with significantly altered expression following chronic and combined acute stress were unique to each strain and represented very different underlying biological processes. Similar findings were also reported for males of the B and D strains following exposure to a more severe stress paradigm (chronic restraint stress followed by forced swim) (Mozhui et al., 2010). The exceptions to this trend were a handful of transcribed genes whose patterns of expression reflected up-regulation (*Nhlh2, Gimap6*, and *Igkv4-58*) or down-regulation (*Fkbp5, Fn1, Fmo2, Mertk, Gkn3, Dbp, Hif3a, Tmc7, Paqr5, Ptprb, Ly6c1*, and *Ly6c2*) within two or more strains (B and D, or N and D). These genes may represent common biological pathways associated with adaptation to chronic stress exposure. For example, *Nhlh2I, Hif3a*, and *Fmo2* are involved in the general response to environmental stress (Heidbreder et al., 2003; Vella et al., 2007; Shetty et al., 2017). *Ly6c1, Ly6c2, Gimap6, Igkv4-58, Fn1, Mertk*, and *Gkn3* are involved in immune response (Menheniott et al., 2010; Ji et al., 2013; Pascall et al., 2013; Mohle et al., 2016; Ramirez et al., 2016), and *Fkbp5* is involved in the inhibition of glucocorticoid receptor function; (Wochnik et al., 2005). Finally, *Dbp, Paqr5, Ptprb*, and *Tmc7* are involved in the regulation of circadian rhythm (Lopez-Molina et al., 1997), progesterone signaling (Petersen et al., 2013), repression of EGFR signaling pathways (Yao et al., 2017), and psychosis risk (Ortega-Alonso et al., 2017), respectively. In contrast, unique transcripts with stress responsive signaling in the B strain were enriched for behavior and excitatory synaptic signaling while transcripts in the D strain were enriched for a number of biological processes, including cell adhesion, RNA splicing, regulation of protein modifications, and lipid localization. Transcripts unique to the N strain were enriched for sensory perception of smell. Thus, stress dependent regulation of the HPA-axis is likely to occur through a number of different pathways both independent of, and dependent on, genotype.

Nearly all of the common transcripts detected between strains demonstrated opposite (increased vs. decreased) expression between B and N substrains and the D strain. Bioinformatic analysis revealed that stress responsive transcripts with increased expression in B and decreased expression in D following chronic stress and exposure to a novel acute stress challenge were enriched for synaptic localization (*Asic1, Pcdh15, Grm8, Prkar2b, Lrrtm3*, and *Olfm2*). This finding suggests that these genes act in a genotype dependent manner to modulate hippocampal neuronal excitability in response to stress experience. In particular, *Grm8* (*mGluR8*; metabotrobic glutamate receptor 8) is a presynaptic autoreceptor (Ferraguti and Shigemoto, 2006) that modulates glutamatergic signaling via negative feedback loops. *Grm8* is expressed in a number of areas involved in stress response, including the hippocampal formation (Goddyn et al., 2015), and is likely to play an important role in the integration and regulation of neuronal excitability and stress response (Linden et al., 2003). This has been demonstrated in the bed nucleus of the stria terminalis – a key direct negative regulator of PVN excitability (Gosnell et al., 2011). Both *Grm8* and *Asic1* are thought to play a role in modulating stress-related emotional traits such as fear and anxiety. Genetic deletion of *Grm8* is also associated with alterations in fear- and anxiety-like behavior (Fendt et al., 2010). *Asic1* (acid-sensing ion channel 1a) is a chemosensor that detects both acidosis and elevated levels of carbon dioxide and has been shown to mediate carbon dioxide-induced fear (Chu and Xiong, 2012). Genetic deletion of *Asic1*, is also associated with increased presynaptic release probability in hippocampal neurons (Cho and Askwith, 2008) and deficits in fear conditioned learning (Wemmie et al., 2002, 2003). In contrast, overexpression of *Asic1* results in enhanced fear learning (Wemmie et al., 2004).

Less is known about the possible role of *Lrrtm3* and *Pcdh15* in the response to stress. *Lrrtm3* is a postsynaptic adhesion molecule that has been shown to be essential for the development of excitatory synapses in dentate gyrus granule neurons (Um et al., 2016). Protocadherins are cellular adhesion molecules implicated in synapse development. The expression of protocadherin *Pcdh15*, was found to be associated with variation in serotonin transporter and serotonin levels in midbrain and hippocampus (Ye et al., 2014). Associations between stress, serotonin imbalance, and risk of stress-related disorders such as depression are often reported. However, serotonin levels were not measured in this study and the role of *Pcdh15* in hippocampus and a potential link to stress-induced modulation of serotonin levels has yet to be investigated.

The biological function of genes with differential hippocampal expression between the N and D strains in response to stress was less clear. These genes, including *Srsf5, Snrnp70, Son, Zfc3h1, Tia1, Rnpc3, Luc7l3*, and *Srsf6*, were enriched for RNA regulation, splicing, processing, and localization. Their expression was increased in N and decreased in D following an acute stress challenge and previous stress experience. These results suggest a decrease in RNA processing associated with stress in the D strain relative to the N strain but the implication of these differences remains to be investigated.

### Summary and Future Directions

Taken together, our findings reveal unique systems level responses to chronic stress in females that vary based on genotype. Identification of the genetic factors and systems level interactions driving these differences is critical for identifying susceptible populations and treatment and prevention of the adverse behavioral and health consequences associated with chronic stress. Importantly, our exploratory analysis provides the genetic framework on which to construct larger mechanistic systems genetics analyses of stress response. Specifically, we have demonstrated that genetic variation among the highly divergent B and D strains, and even among the highly similar B and N substrains, is responsible for differences in the physiological response to acute and chronic stress. The major implication of these findings is that repetition and expansion of our study in the BXD population derived from the B and D strains, or in a reduced complexity cross between B and N substrains will identify the genetic drivers of the physiological response to stress. Reduced complexity crosses are useful for rapid identification of gene variants with a large impact on trait variation and recombinant inbred panels, such as the BXD panel, are the superior choice for systems level analyses due to the ability to resample genotypes and acquire dense multi-scalar data for each member of the population. Importantly, these rodent genetic populations can be used to identify the precise genetic and molecular drivers of the physiological and behavioral response to stress. These systems offer three main advantages to human linkage or genome-wide association studies: (1) design and control of environmental manipulations, (2) the ability to manipulate the genome, and (3) accumulation of multi-scalar data from brain gene expression to physiological and behavioral response that are required for systems level analyses. In contrast to human studies, rodent genetic systems offer an unparalleled opportunity to understand interactions between genes and environmental exposures that contribute to organism response. In addition, these systems can be reprogrammed to incorporate output from human genetic studies in order to provide a better understanding of the collective contribution of gene variants to disease (Neuner et al., 2019).

## Ethics Statement

This study was carried out in accordance with the recommendations of the University of Tennessee Health Science Center Institutional Animal Care and Use Committee. The protocol (Institutional Protocol 14–131) was approved by the University of Tennessee Health Science Center Institutional Animal Care and Use Committee.

## Author Contributions

ET, SC, PM, BJ, MM, and LL designed the study. ET, PM, BJ, WZ, and MM performed the experiments. ET, SC, PM, CP, BJ, and MM analyzed the data. ET, MM, and BJ did the majority of the writing.

## Conflict of Interest Statement

The authors declare that the research was conducted in the absence of any commercial or financial relationships that could be construed as a potential conflict of interest.

## Footnotes

1 http://www.genenetwork.org/webqtl/main.py?FormID=sharinginfo“&GN“_AccessionId=771; GN Accession GN771

## Supplementary Material

FIGURE S1 CMS Methods. Daily treatment schedule is shown. The same schedule was repeated each week for 7 weeks.

FIGURE S2 Fecal and Plasma CORT Levels at Time of Tissue Harvest. **(A)** Baseline fecal CORT levels are shown for each strain. The D strain has high basal fecal CORT. **(B)** Plasma CORT levels (NH and CMS groups) were collected at tissue harvest and provide baseline (NH) and post-CMS estimates of circulating CORT levels. Plasma CORT levels are significantly higher in CMS treated mice. CMS is associated with significantly higher plasma CORT in the B strain and there is a trend for higher CORT associated with CMS in the D strain.

TABLE S1 Summary of subjects by strain and treatment.

TABLE S2 Body and tissue weight correlations.

TABLE S3 List of transcripts with significant strain or treatment effects.

TABLE S4 Enrichment analysis results for overlapping transcripts among strains.

TABLE S5 Enrichment analysis results for strain-specific transcripts.
