# Supplementary figures and images for: Genetic Factors Mediate the Impact of Chronic Stress and Subsequent Response to Novel Acute Stress
Source: Front Neurosci. 2019 May 21;13:438. doi: 10.3389/fnins.2019.00438 (PMC6536627; doi:10.3389/fnins.2019.00438)

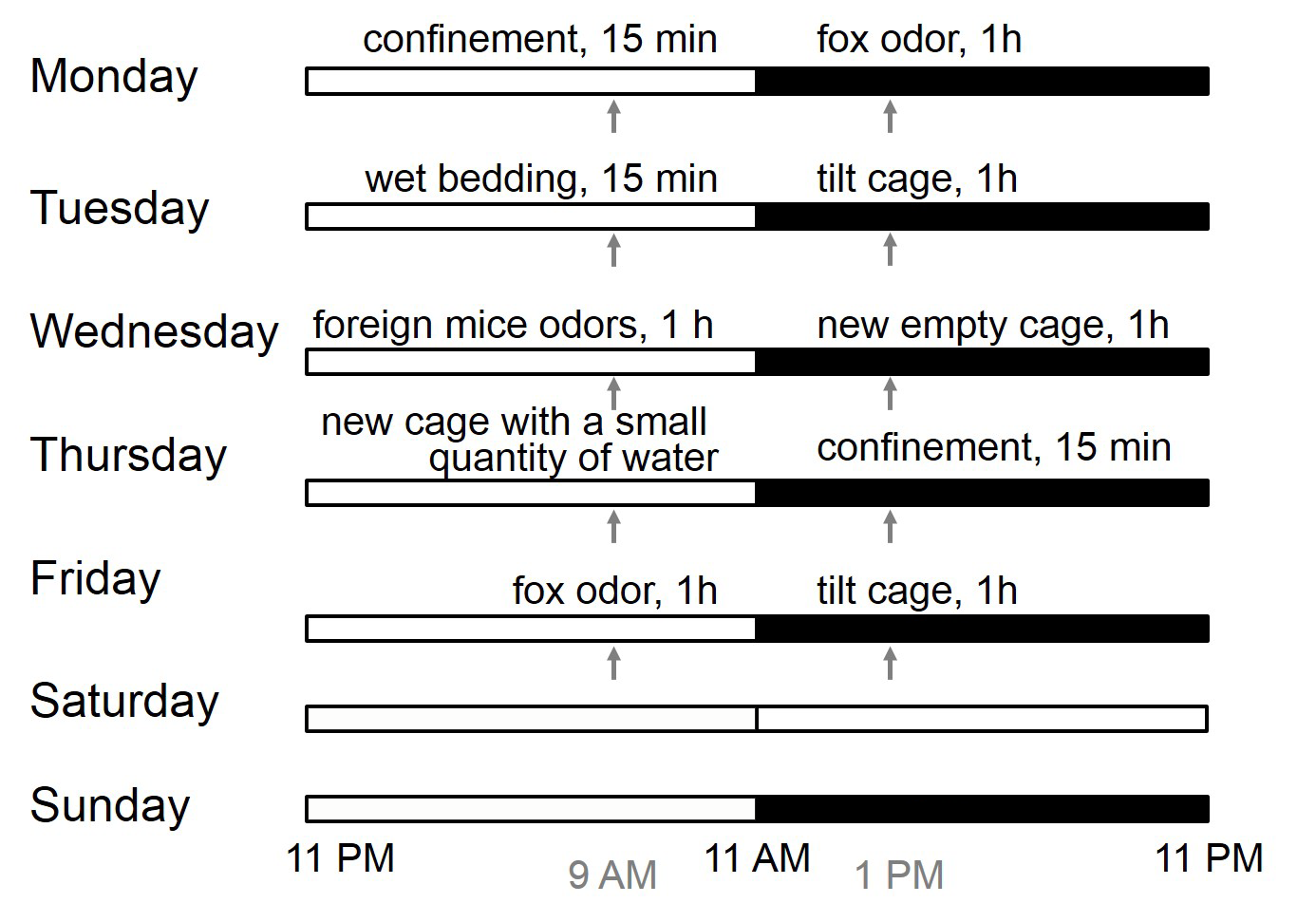

Supplement: FIGURE S1 — CMS Methods. Daily treatment schedule is shown. The same schedule was repeated each week for 7 weeks. [file Image_1.TIF]

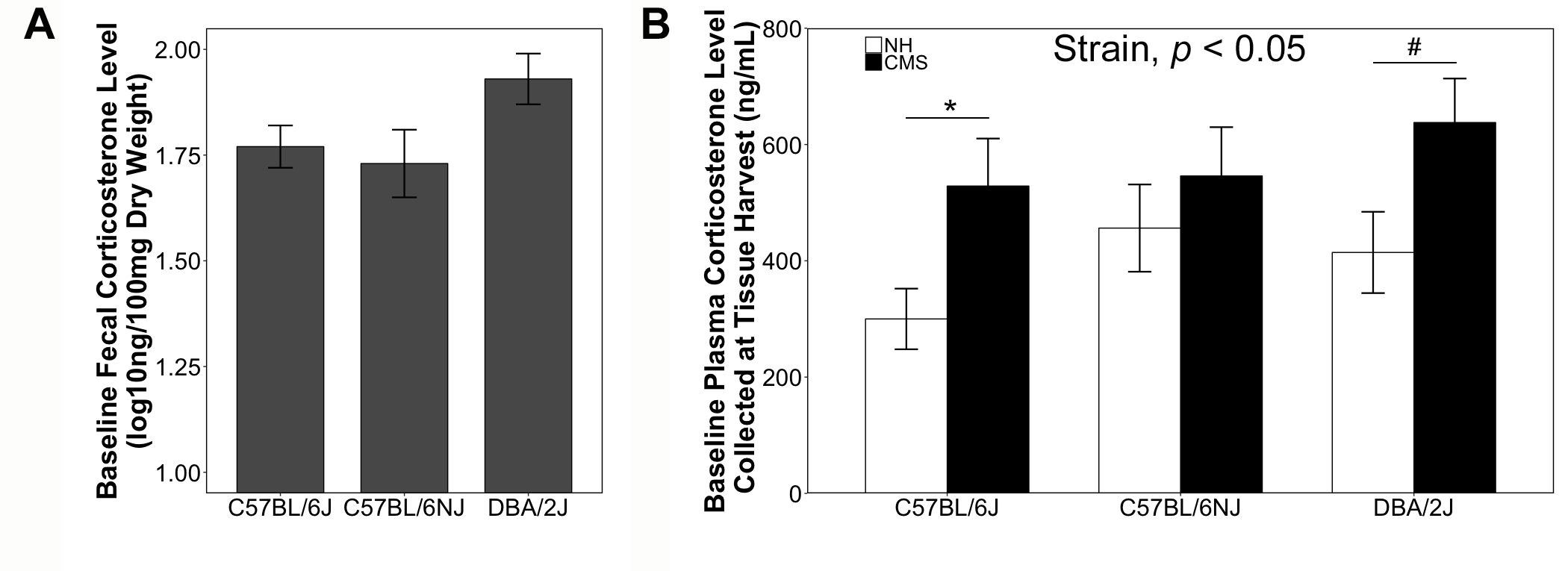

Supplement: FIGURE S2 — Fecal and Plasma CORT Levels at Time of Tissue Harvest. (A) Baseline fecal CORT levels are shown for each strain. The D strain has high basal fecal CORT. (B) Plasma CORT levels (NH and CMS groups) were collected at tissue harvest and provide baseline (NH) and post-CMS estimates of circulating CORT levels. Plasma CORT levels are significantly higher in CMS treated mice. CMS is associated with significantly higher plasma CORT in the B strain and there is a trend for higher CORT associated with CMS in the D strain. [file Image_2.TIF]
